# Growth mechanism of epitaxial YSZ on Si by Pulsed Laser Deposition

**DOI:** 10.1038/s41598-018-24025-7

**Published:** 2018-04-10

**Authors:** David Dubbink, Gertjan Koster, Guus Rijnders

**Affiliations:** MESA+ Institute for Nanotechnology, University of Twente, Enschede, The Netherlands

## Abstract

The epitaxial growth of yttria-stabilized zirconia (YSZ) on silicon with native oxide was investigated in order to gain more insight in the growth mechanism. Specifically, attention was paid to the possibilities to control the chemical interactions between YSZ, silicon and oxygen during initial growth. The sources of oxygen during growth proved to play an important role in the growth process, as shown by individual manipulation of all sources present during Pulsed Laser Deposition. Partial oxidation of the YSZ plasma and sufficient delivery of oxygen to the growing film were necessary to prevent silicide formation and obtain optimal YSZ crystalline qualities. In these conditions, thickness increase of the silicon native oxide before growth just started to occur, while a much faster regrowth of silicon oxide at the YSZ-Si interface occurred during growth. Control of all these contributions to the growth process is necessary to obtain reproducible growth of high quality YSZ.

## Introduction

In order to bring single crystalline oxide thin films towards commercial applications, epitaxial integration on silicon wafers is required. Epitaxial integration of oxides on silicon is a challenging task, mainly because of the chemical interactions between silicon, oxygen and metal oxides. Most of the metal oxides react with silicon to form silicide and/or silicate phases^1,2^, which prevents epitaxial crystallization of the growing oxide and can deteriorate the functional properties of the film. Besides, an amorphous native oxide always forms on silicon, preventing growth of the oxide directly on the silicon crystal lattice. This native oxide can be removed prior to growth, but ultra high vacuum conditions are required to keep the very reactive bare silicon surface free from carbide and oxide formation^3,4^. Those conditions are hard to reach in growth systems, and low oxygen pressures are contrary to the necessity to supply sufficient oxygen to the growing oxide film^5^. In order to avoid these issues, yttria-stabilized zirconia (YSZ) can be used as a buffer layer to incorporate epitaxial oxides on Si. During growth in reducing conditions, the deposited YSZ decomposes the native oxide through redox reactions, after which a chemically stable film crystallizes epitaxially on the Si crystal lattice^6^. In this way, formation of unstable surfaces is avoided, and therefore the need to work in ultra high vacuum conditions. Very smooth surfaces can be obtained by a variety of Physical Vapor Deposition techniques, e.g. Pulsed Laser Deposition (PLD)^7–9^, radio-frequency magnetron sputtering^10^ and electron-beam evaporation^6,11^. The highest quality films have a full width at half maximum (FWHM) of the X-ray Diffraction (002) rocking curve of around 0.7°. Normally, an additional epitaxial fluorite CeO_2_ layer is grown on the YSZ film to obtain a suitable template for growth of a perovskite oxide^12^. Growth of epitaxial (001) oriented perovskites with good functional properties on top of these buffer layers is well established^13–16^. However, the growth mechanism of YSZ on Si by e.g. PLD is not known in detail, which is important to obtain reproducible growth and information about important growth parameters when upscaling growth to large silicon wafers.

Epitaxial YSZ films on Si have been made with PLD since the 90 s. Initially, YSZ films were grown on Si with the native oxide removed in advance^7^. Growth on silicon with native oxide was introduced short time later^17^, and appeared to deliver films with higher crystalline qualities^6,10,18^. Typically, a two step growth process is used^8,13^. The first couple of nm are deposited in low oxygen pressure to perform a scavenging reaction. ZrO_2_ and Y_2_O_3_ have lower Gibbs free energies of formation compared to SiO_2_. Therefore, Zr and Y will scavenge the oxygen from the silicon oxide when brought into contact with silicon oxide in low oxygen pressures^17,19^. The decomposition of the native oxide occurs most probably by formation of volatile SiO, corresponding to the following reaction^6^:

1 $$Zr+2Si{O}_{2}\to 2SiO\uparrow +Zr{O}_{2}$$

The used pressures during growth with PLD vary from base pressure (often in the 10^−7^ mbar range) to 10^−4^ mbar O_2_. Crystallization of YSZ on the silicon crystal lattice is typically observed after deposition of about 1 nm^6,9^. In the second step, after deposition of about 5 nm, more oxygen is added to fully oxidize the film during the remainder of the growth. Control of the chemistry during the first step seems to be critical for the crystalline quality of the resulting film. Deposition of too large amounts of YSZ in reducing conditions leads to formation of silicides, which increases the amount of defects in the YSZ film^20^. Furthermore, the presence of residual native oxide may aid the crystallization of YSZ by avoiding the large lattice mismatch between Si and YSZ (5.7%), either via lateral overgrowth^20^ or via crystallization on the crystalline part of the native oxide, which may be situated close to the silicon surface with lattice parameters closer to YSZ^18,21^.

The work described above shows the occurance and importance of several chemical processes during initial growth of YSZ on Si by PLD, e.g. silicon oxide reduction and silicide formation. However, limited attention has been paid to the possibilities to control these different chemical processes. An unique feature of the PLD process is the interaction of the plasma with the background gasses present in the deposition chamber. The metals in the plasma can obtain different degrees of oxidation depending on the partial oxygen pressure^22^. As shown for the homoepitaxial growth of SrTiO_3_ (STO), stoichiometry and growth kinetics depend heavily on the degree of oxidation of the plasma. Furthermore, the STO substrate proved to supply oxygen to the growing STO film as well^23^. Similar to the growth of STO on STO, three sources of oxygen can be distinghuised during growth of YSZ on Si with native oxide. At the substrate/film surface, oxygen can arrive from the plasma as atomic or molecular oxygen, or in the form of (partially) oxidized zirconium and yttrium. The oxygen from the background can oxidize the growing film directly, but also interact with the plasma. Furthermore, oxygen is present in the silicon native oxide. The thickness of this oxide can change during heating to the YSZ growth temperature due to reaction with oxygen from the background. Since oxygen is involved in all of the chemical processes described before, tuning the contributions of all sources of oxygen may provide a way to control the chemistry during the scavenging process.

In this work, the possibility to control the chemistry of the initial growth of YSZ on Si was investigated, as well as the relationship between the chemistry and the resulting crystalline properties of the YSZ film. Both subjects were assessed by detailed study of the PLD growth process, with a focus on the contributions of the different sources of oxygen. In order to investigate these contributions, all sources were adressed individually:

1. **Background pressure**. The contribution of oxygen from the background was varied by changing the partial oxygen pressure (pO_2_) at constant total pressures. Ar was used to reach the total pressure aimed for, since it is inert and has an atomic weight close to the weight of molecular oxygen. In this way, the plasma plume size and shape was kept similar, meaning the flux of oxygen from the background could be changed independently from the Zr and Y fluxes from the plasma. Additionally, the fluxes of Zr and Y from the plasma could be changed independently by changing the laser repetition rate.
2. **Plasma**. The physics and chemistry of the plasma changes drastically with pressure^22,24^. For instance, the oxidation state of the plasma upon arriving at the substrate can be different for similar pO_2_, while the total pressure influences the arrival time and plasma temperature. For this reason, 2 different total pressures were examined, i.e. 2*10^−2^ and 1*10^−1^ mbar. The resulting physics and chemistry of the plasma were examined with self-emission spectroscopy.
3. **Native oxide**. All 5 × 5 mm Si substrates were cut from the same 4 inch wafer in order to start with the same native oxide thicknesses in all experiments. However, the thickness can change due to heating of the substrate in the presence of oxygen. Therefore, *in situ* X-ray Photoelectron Spectroscopy (XPS) was used to determine silicon oxide thicknesses of the substrates in different deposition conditions.

The results section is divided into two parts. First, chemical and crystallization processes observed during initial growth in the different conditions are described, as well as the resulting crystalline properties. Reflection High-Energy Electron Diffraction (RHEED) was used to monitor the crystallization process during growth. In order to investigate the chemistry after growth, *in situ* X-ray Photoelectron Spectroscopy (XPS) was used. X-ray Diffraction (XRD) and Atomic Force Microscopy (AFM) were used to relate the observed growth processes to respectively the crystalline properties and morphology of the films. Secondly, the contributions of the different sources of oxygen to those chemical and crystallization processes were investigated, as described above.

## Results

### Correlation between initial growth and crystalline quality

**Figure 1:**
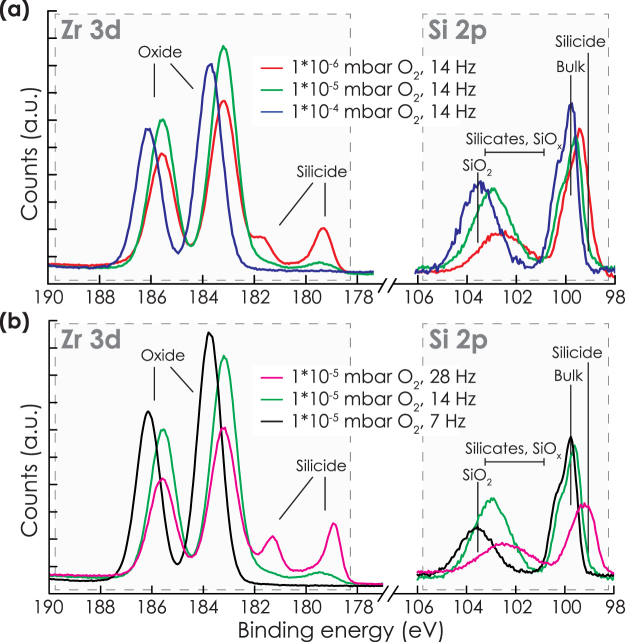
(**a**) XPS Zr3d and Si2p spectra of films grown at total pressures of 2*10^−2^ mbar at (**a**) different pO_2_ or (**b**) with different laser repetition rates. A low flux of oxygen compared to Zr, caused by low pO_2_ or high laser repetition rate, led to silicide formation and an increased ratio of silicate/SiO_*x*_ to SiO_2_ bonds.

First, the influence of pO_2_ on the chemical interactions during initial growth was investigated. Figure 1a shows XPS spectra of 6 nm YSZ films grown at different pO_2_, while the total pressure and laser repetition rate were kept constant at respectively 2*10^−2^ mbar Ar and 14 Hz. Zr silicide formation was clearly observed when a pO_2_ of 1*10^−6^ mbar was used, as concluded from the existence of Zr^0^ peaks together with a shoulder at the low binding energy side of the Si2p bulk peak^25^. The intensities of these features were lower at 1*10^−5^ mbar, and were completely absent when pO_2_ of 1*10^−4^ mbar or higher were used. A similar change was obtained by changing the flux of Zr and Y using different laser repetition rates. The XPS spectra in Fig. 1b show that silicide formation decreased when the laser repetition rate was decreased from 28 to 14 Hz at a constant pO_2_ of 1*10^−5^ mbar, whereas no silicide formation was detected anymore at 7 Hz. Thus, the formation of Zr silicides can be controlled by tuning the ratio between flux of oxygen from the background gas and Zr and Y from the plasma. Although similar trends can be expected for Y, the changes in binding energies are too small to clearly identify the different phases (see Supplementary Fig. S1).

A second notable difference appeared in the Si2p region indicating oxidized species. In the Si2p region, peaks around 99.7 eV and103.5 eV indicate the Si^0^ substrate and completely oxidized Si^4+^ respectively. In between both extremes, underoxidized Si (SiO_*x*_) and/or silicates (Y/Zr-O-Si) can appear^26^. In principle, at least one monolayer of silicate bonds is expected due to the interface between YSZ and Si or SiO_2_, which contributes significantly to the XPS spectra due to the surface sensitivity of XPS. As visible in Fig. 1, the region indicating SiO_2_ increased with respect to the region indicating silicates and SiO_*x*_ species when the pO_2_ was increased or the laser repetition rate was decreased. Although any quantification cannot be performed without knowledge about the morphology and distribution of the different species, the measurements suggest increasing regrowth of SiO_2_ with increasing pO_2_ or decreasing laser repetition rate. Quantification of the SiO_2_ thickness will be discussed later.

**Figure 2:**
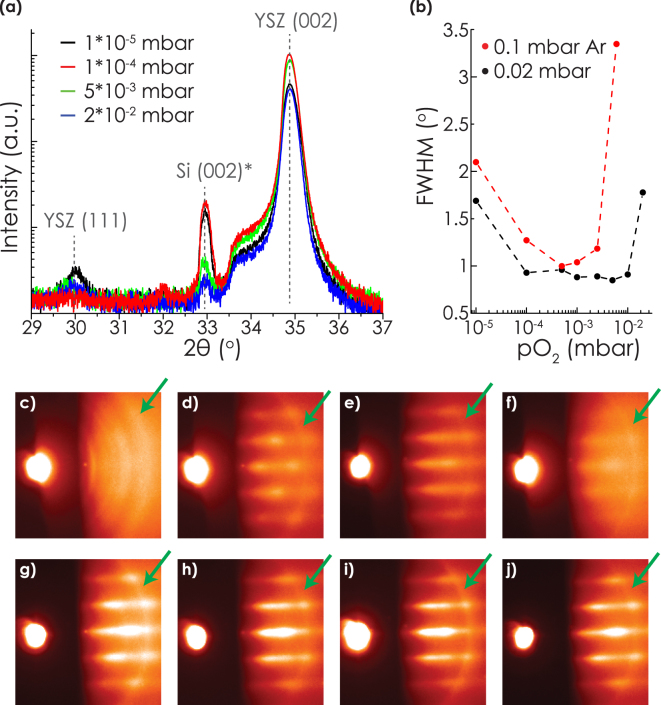
XRD measurements of 100 nm thick YSZ films on top of 5 nm YSZ films grown in varying pressures. (**a**) XRD *θ* − 2*θ* scans of the films with the first 5 nm grown at a total pressure of 2*10^−2^ mbar Ar. At 33° a multiple reflection peak of the Si can be observed. The variation in intensity of this peak is only related to the in-plane orientation of the sample in the XRD^40^. (**b**) FWHM of the YSZ (002) rocking curves of the samples with the initial 5 nm grown in total pressures of 2*10^−2^ or 1*10^−1^ mbar. The dashed lines are inserted for visual reference. (**c**–**f**) RHEED images corresponding to the films shown in subfigure a, after growth of 5 nm in respectively 1*10^−5^, 1*10^−4^, 5*10^−3^ and 2*0^−2^ mbar. The images were taken after adding the 0.02 mbar O_2_ to the growth chamber for growth of the 100 nm layer. (**g**–**i**) RHEED images of the same samples after growth of the 100 nm layer in 0.02 mbar O_2_. The rings indicated by a green arrow are artefacts formed by reflection of the electron beam at its entrance pinhole to the growth chamber.

In order to investigate the influence of the initial chemistry on the crystalline properties of YSZ, 100 nm YSZ films were grown on top of 5 nm films which were grown under varying pO_2_ and a fixed laser repetition rate of 14 Hz. This laser repetition rate was chosen because of the clear oxygen pressure dependent differences in chemistry during initial growth, as shown by the XPS measurements above. The 100 nm films were all grown with the same depostion conditions (p = 2 $\ast$ 10^−2^ mbar O_2_, f = 14 Hz). In this way, XRD measurements of the thick films acted as a tool to indicate the crystalline properties of the first 5 nm. The XRD measurements presented in Fig. 2 show a clear trend in crystalline properties depending on the pO_2_ at 2*10^−2^ mbar Ar during initial growth. At low pO_2_, (111) oriented YSZ was measured besides the epitaxial (001) orientation (besides the RHEED patterns shown in Fig. 2, see Supplementary Fig. S2 for a *ϕ*-scan confirming the epitaxial relation between Si and YSZ). The intensity of the (111) peak decreased with increasing pO_2_. At the same time, the FWHM of the rocking curve of the (002) peak decreased. At a pO_2_ of 5*10^−3^ mbar, the lowest FWHM was measured, while no (111) orientation was visible anymore. Increasing the pO_2_ above this value led to increased values of the FWHM and the presence of (111) oriented YSZ again.

The corresponding RHEED patterns revealed similar information. Rings were observed after growth of 5 nm in 1*10^−5^ mbar O_2_, indicating the presence of polycrystalline phases (see Fig. 2c). The presence of polycrystalline phases was consistent with the presence of the (111) orientation measured with XRD. Other orientations were hardly visible in the XRD spectra due to the low relative intensities of these peaks. Typically, streaks, indicating a flat surface, appeared when the FWHM of the XRD (002) rocking curve was below 1° (see Fig. 2e), while spots, indicating a rougher surface, appeared above this value (Fig. 2d,f). In all cases, the sharpness of the RHEED spots or streaks increased after growth of the 100 film YSZ film (see Fig. 2g–j), indicating improved surface crystallinity. Even in the case rings were observed after growth of the 5 nm film, a well-defined pattern evolved gradually during growth of the 100 nm film (see Fig. 2g).

A similar trend was observed when the initial growth was performed at a total pressure of 1*10^−1^ mbar (see Fig. 2b). The growth rate per second was kept similar to the 2*10^−2^ mbar experiments by using a laser repetition rate of 12.5 Hz. Despite the equal growth rate, the lowest FWHM was observed at 5*10^−4^ mbar, which is one order of magnitude lower compared to the growth performed at a total pressure of 2*10^−2^ mbar. The lowest FWHM was 1.00°, while an optimum of 0.85° was obtained in the 2*10^−2^ mbar case. Furthermore, features indicating polycrystallinity started to dominate the RHEED pattern at 5*10^−3^ mbar, while streaks or spots indicating epitaxial phases were not observed at all at a pO_2_ of 2*10^−2^ mbar. This degree of polycrystallinity differed from the growth performed at a total pressure of 2*10^−2^ mbar, since only a small amount of polycrystalline phases was observed with XRD when 2*10^−2^ mbar O_2_ was used (see Fig. 2a).

**Figure 3:**
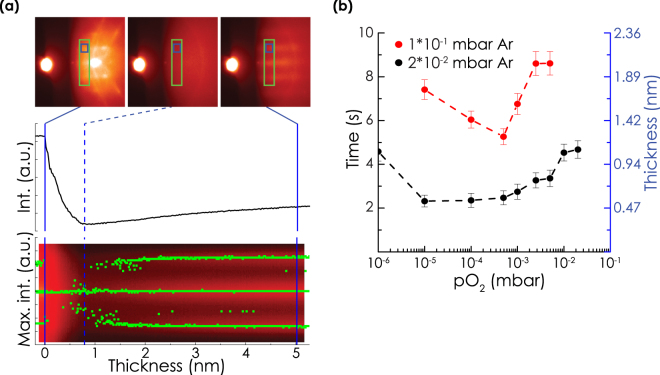
(**a**) Snapshots from a RHEED movie recorded during growth at 5*10^−3^ mbar O_2_ in a total pressure of 2*10^−2^ mbar Ar. Below the snapshots, the intensity profile derived from the blue box and the streak positions derived from the green box are presented. The dashed line indicates the minimum intensity, which is taken as a measure of the crystallization time/thickness. (**b**) Crystallization times/thicknesses derived with the method depicted in subfigure (**a**), for samples grown at different pO_2_ in total presures of 2*10^−2^ and 1*10^−1^ mbar Ar. The dashed lines are a guide to the eye.

During growth of the films, RHEED movies were recorded with ~0.1 frame/s in order to obtain insights about the crystallization behavior in the different growth conditions. Figure 3a presents an example of the analysis performed on the RHEED data. Before start of the growth, the pattern of the crystalline surface buried beneath the amorphous silicon oxide was visible. This pattern faded when YSZ was deposited due to increased attenuation by the deposited material. After a certain deposition time, streaks or spots indicating epitaxial YSZ appeared. The intensity of a disappearing Si spot and evolving YSZ streak or spot was monitored over time. The minimum intensity was used as an indication of the crystallization time. This crystallization time serves as an indication of the efficiency of the native oxide decomposition and the following YSZ crystallization, and therefore reveals important details about the YSZ growth mechanism, as will be described in more detail in the discussion section. Figure 3b shows the crystallization times as well as the corresponding amount of deposited YSZ, determined for samples grown at different pO_2_ and total pressures of 2*10^−2^ or 1*10^−1^ mbar Ar. Similar trends were visible in the crystallization time for both total pressures. First the crystallization time decreased with increasing pO_2_, after which it increased again. At a total pressure of 1*10^−1^ mbar, the minimum in crystallization time was observed at the same pO_2_ as the optimum crystalline quality (see Fig. 2). At the total pressure of 2*10^−2^ mbar, a minumum was observed at a pO_2_ of 1*10^−5^ mbar, after which the crystallization time slightly increased. Crystallization times were notably larger at 1*10^−1^ mbar.

Figure 3a shows a typical example of the change of the positions of the Si and YSZ streaks with increasing deposition time. No change in the distance between the YSZ streaks was observed above 2 nm, even after growth of additional 100 nm in oxygen atmosphere. The distance between the YSZ streaks was larger compared to Si, indicating a smaller lattice, as expected. Below 2 nm, the YSZ streak positions were closer to the Si streak positions. Although this may indicate that YSZ was initially strained to the silicon, the shift can be caused by overlap with the Si streaks, which were still weakly present at the starting point of YSZ crystallization. Similar fast lattice relaxation during growth was observed for all films.

### Contribution of sources of oxygen to the growth process

**Figure 4:**
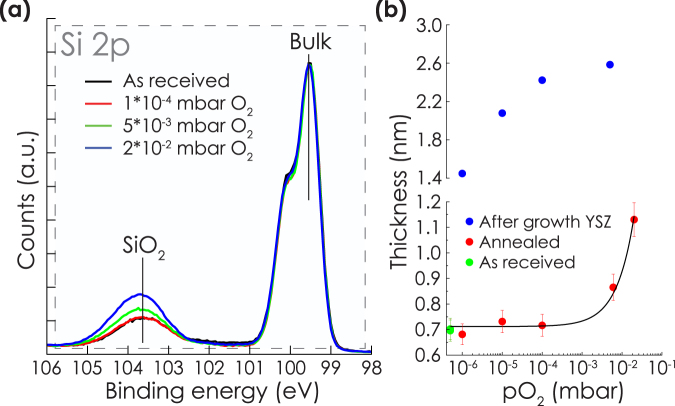
(**a**) XPS Si2p spectra of Si substrates after heating to 800 °C at different pO_2_. Increase of oxide thickness was observed above 1*10^−4^ mbar. (**b**) Calculated silicon oxide thicknesses for as received and annealed substrates, and after growth of YSZ. The silicon oxide thicknesses for the samples with YSZ film were calculated from the samples shown in Fig. 1a, i.e. 6 nm YSZ films grown at a total pressure of 2*10^−2^ mbar with a laser repetition rate of 14 Hz. The black line is a linear fit of the data..

Figure 4a shows the Si2p spectra for silicon substrates with native oxide after annealing at 800 °C for 5 minutes at different pO_2_. An increase in the intensity of SiO_2_ with respect to Si from the bulk of the substrate was notable above a pO_2_ of 1*10^−4^ mbar. Using the model described in the methods section, the silicon oxide thicknesses were calculated from these spectra. A linear increase of oxide thickness with pO_2_ was observed, as shown in Fig. 4b. Growth of YSZ was normally started within 30 seconds after reaching 800 °C. This is especially important for the higher pO_2_, since the thickness of the silicon oxide is expected to increase approximately linearly with time^27^. For example, when growth is performed at a total pressures of 2*10^−2^ mbar O_2_, the increase in oxide thicknes is expected to be only 0.04 nm when the substrate is kept at 800 °C for 30 s, instead of the observed 0.4 nm when the substrate is kept at 800 °C for 5 min. Indeed, polycrystalline growth was observed in the latter case (data not shown), while epitaxial growth was observed when the growth started immediately after reaching 800 °C (see Fig. 2).

Secondly, the SiO_2_ thicknesses after growth of YSZ were calculated for the samples grown in a total pressure of 2*10^−2^ mbar. In order to perform the calculation, a homogeneous Si-SiO_2_-YSZ stacking sequence was assumed. After fitting, only the parts of the spectra indicating SiO_2_ and Si were taken into account by subtracting the silicide, silicate and SiO_*x*_ contributions, which were especially present in the low pO_2_ samples. A significant increase in silicon oxide thickness with increasing pO_2_ was calculated, as shown in Fig. 4b. Although the samples were cooled down in vacuum directly after growth, the oxide thicknesses were much larger compared to the bare substrates annealed at the same pO_2_. In the case of growth at pO_2_ = 5*10^−3^ mbar, where an optimum crystalline quality was observed with XRD, a silicon oxide thickness of 2.6 nm was determined.

**Figure 5:**
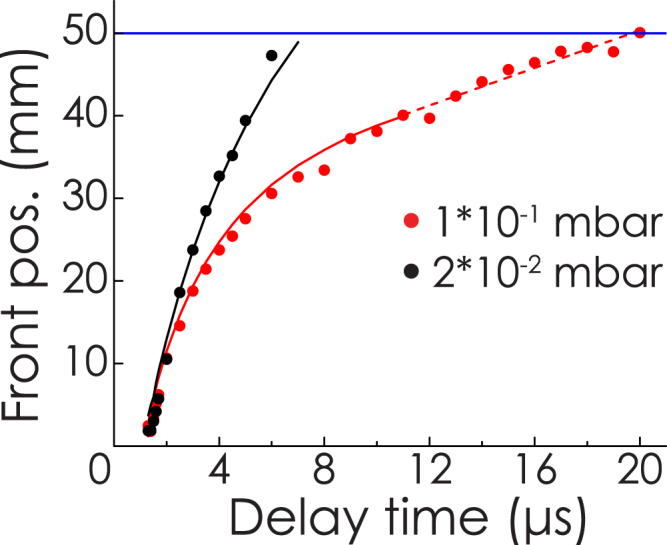
Plot of plasma front positions versus delay times at 2*10^−2^ and 1*10^−1^ mbar O_2_. The solid lines represent a drag model fit, the dashed curve is a linear fit indicating diffusive propagation. The blue line is the substrate position.

The chemistry and kinetics of the plasma were investigated for different pO_2_ at total pressures of 2*10^−2^ and 1*10^−1^ mbar. Figure 5 presents the front position versus delay time of the plasma plume at pressures of 2*10^−2^ and 1*10^−1^ mbar O_2_. The plasmas propagated differently in both pressures. At 2*10^−2^ mbar, the plasma arrived at the substrate 6 *μ*s after ablation, with a velocity of 5 km/s. The propagation could be fitted with a simple kinetic drag model^28^. In this model, the plasma has a ballistic like propagation, while minor deceleration occurs due to drag forces on the particles in the plasma. At 1*10^−1^ mbar, the plasma front position changed linearly with delay time after 12 *μ*s, which indicates propagation by diffusion. The plasma reached the substrate after 20 *μ*s with a velocity of 1 km/s. The plasma propagation behavior and arriving velocities were very similar to other oxide plasmas^24^.

**Figure 6:**
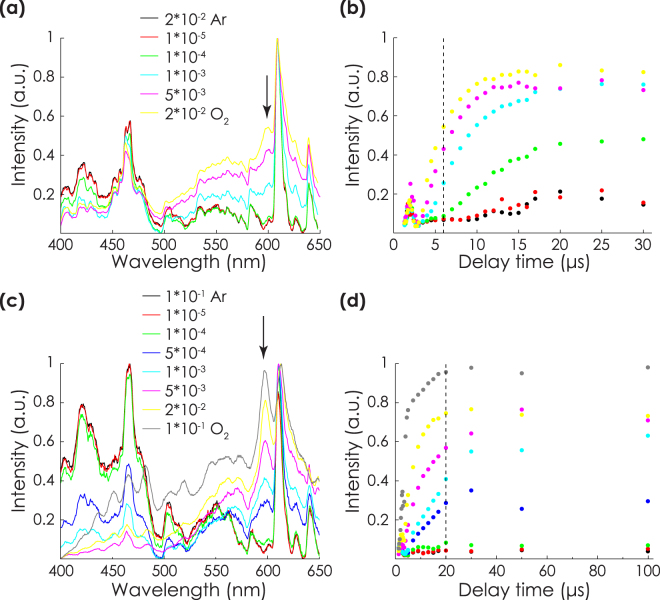
Self emission spectra of the plasma at total pressures of (**a**) 2*10^−2^ and (**c**) 1*10^−1^ mbar after arriving at the substrate, respectively 6 and 20 *μ*s after ablation. The YO line at 597 nm is indicated with a black arrow. (**b**,**d**) Show the relative intensities of the YO line at 597 nm after different delay times for total pressures of 2*10^−2^ and 1*10^−1^ mbar respectively. The color scales correspond to subfigures (**a**,**c**), the arrivial of the plasma plume at the substrate is indicated with a dashed line.

Figure 6a,c shows the spectra of the plasmas at different pO_2_ just after arriving at the substrate. The spectra show a clear trend depending on pO_2_ for both 2*10^−2^ and 1*10^−1^ mbar total pressures. Comparison with spectra from the binary oxides and reference tables^29,30^ showed that the plasmas were dominated by atomic Zr lines at low pO_2_. Especially, the lines between 400 and 500 nm can be assigned to atomic Zr species. The relative intensity in this region decreased with increasing pO_2_. Simultaneously, between 500 and 600 nm bands originating from zirconia increased. With increasing oxidation, the contribution of yttria to the spectra became stronger as well. Especially, a strong YO band showed up at 597 nm. In order to compare the oxidation of the plasmas, the relative intensity of this line was determined for all pO_2_ after different delay times. Figure 6b,d show the results for the total pressures of 2*10^−2^ and 1*10^−1^ mbar respectively. The presence of the YO line was noted above pO_2_ of 1*10^−4^ mbar in 2*10^−2^ mbar total pressure, and 5*10^−4^ mbar in the 1*10^−1^ mbar case. When the plasma arrived at the substrate, the YO line was more pronounced at total pressures of 1*10^−1^ mbar, compared to similar pO_2_ in 2*10^−2^ mbar. However, at 2*10^−2^ mbar, the relative intensity of the YO still increased after arrival, while the increase hardly occurred at 1*10^−1^ mbar. For both total pressures, oxidation was observed at pO_2_ corresponding to optimal YSZ quality (5*10^−3^ and 5*10^−4^ for 0.02 and 1*10^−1^ mbar respectively, see Fig. 2).

## Discussion

A high ratio of Y/Zr to oxygen during growth caused formation of silicides, which could be tuned by changing the pO_2_ and the laser repetition rate. As shown for the case of a laser repetition rate of 14 Hz and a total pressure of 2*10^−2^ mbar, silicide formation occurred at pO_2_ below 1*10^−5^ mbar. At these conditions, plasma spectroscopy did not show any oxidation of the plasma. Initially, Y and Zr can take sufficient oxygen from the native oxide to form YSZ. However, oxygen deficiency may occur due to ongoing deposition of metal atoms. Oxygen deficiency makes YSZ unstable towards silicide formation when contacted with silicon^31^. In a pO_2_ of 1*10^−5^ mbar, the thermodynamically expected amount of vacancies is much lower than the amount causing instability^31^, while the flux of oxygen from the ambient should be sufficient to provide the necessary oxygen for complete oxidation of the growing film, considering the used growth speed (~0.5 unit cells per second at 14 Hz). Apparently, excess of O_2_ at the film surface is necessary to completely oxidize the film during growth.

Silicide formation explains the trend in the crystallization time at low pO_2_. In principle, the scavenging process, followed by YSZ crystallization, should be fastest in the most oxygen deficient conditions, i.e. when Y and Zr do not oxidize in the plasma and regrowth of the silicon oxide due to oxygen from the background gas is limited. Instead, increased crystallization times were observed in the most oxygen deficient conditions, which can be caused by competition between silicide formation and YSZ crystallization at the silicon-YSZ interface. In the higher pO_2_ regime, the crystallization time increased with increasing pO_2_. For both the total pressures of 2*10^−2^ and 1*10^−1^ mbar, the increase occurred in the regime where the plasma started to oxidize. Due to this partial oxididation, the scavenging possibility per Zr or Y atom was lower, meaning more YSZ was needed to break down the silicon native oxide.

Two additional mechanisms were found to contribute to the increased crystallization times. First of all, at pressures above 1*10^−3^, the silicon oxide thickness started to increase before start of the growth. This contribution was largely circumvented by starting the growth quickly after reaching the growth temperature. More importantly, the thickness of the silicon oxide after growth of YSZ depended heavily on the pO_2_, and showed a growth rate much higher than the growth rate on the as received silicon. Similar growth rate enhancement with over an order of magnitude has been described for thin metal overlayers of e.g. Ba^32^, Cu^33^, Sr^34^ and Y^35^. Apparently, YSZ catalyzes the absorption of oxygen by the silicon oxide. Therefore, silicon oxide regrowth is expected to compete with the scavenging process as soon a YSZ is present at the silicon surface. In the case of growth in a total pressure of 2*10^−2^ mbar, the optimum pO_2_ was found at conditions were severe regrowth of the silicon oxide was observed. Together with the observed partial oxidation of the plasma, the scavenging process in optimum conditions can now be summarized by the following reaction:

2 $$Zr{O}_{2-x}+xSi{O}_{2}+{O}_{2}+Si\to xSiO\uparrow +Zr{O}_{2}+Si{O}_{2}$$

If the oxygen pressure is too high, (partly) polycrystalline films grow due to insufficient scavenging power, mainly caused by overoxidation of the plasma and regrowth of the native oxide. Note that the crystallization times correlated well with the crystalline quality of the grown films. In general, low crystallization times led to low FWHM of the YSZ (002) rocking curves (compare Figs 2b and 3b), since both silicide formation and overoxidation of the plasma were avoided.

The observed phenoma agree very well to the lateral overgrowth mechanism proposed by De Coux *et al*.^20^, since regrowth of silicon oxide does not necesseraly prevent lateral crystallization. The observed lattice relaxation from the start of crystallization fits this mechanism as well, and is in agreement with the observations made by Ishigaki *et al*.^18^. Lateral overgrowth is a well known method in growth of semiconductors, and proved to increase the crystalline quality due to avoidance of defect formation because of strain^36,37^. Similarly, the high YSZ crystalline qualities in conditions were residual SiO_2_ is expected, can be explained by this mechanism. As shown in Supplementary Fig. S3, a low surface roughness was obtained as well, as expected for high crystalline quality films. On the other hand, surfaces in silicide forming conditions were much rougher, due to a more local YSZ nucleation and lower crystalline qualities, as shown in more detail in the Supplementary Information (see Supplementary Figs S3–S5).

The trends described above hold in general for both total pressures of 2*10^−2^ mbar and 1*10^−1^ mbar. However, differences where observed in the optimal pO_2_, crystallization times, the obtained crystalline quality and the limiting pO_2_ at which epitaxial growth was not possible anymore. Some aspects of the growth process were similar for both total pressures. The silicon oxide thicknesses at the start of YSZ growth were similar, since the increase in thickness depends on the pO_2_ only. For the same reason, the fluxes of oxygen from the ambient to the growing film were similar. Finally, the flux of YSZ per second was kept constant by using a slightly lower laser repetition rate in the 1*10^−1^ mbar case (12.5 Hz in stead of 14 Hz). Therefore, the differences in growth behavior can only be explained by differences in the behavior of the plasma.

First, differences can be caused by the different kinetic regimes of the plasma. At 2*10^−2^ mbar, the velocity of the plasma arriving at the substrate was much higher compared to 1*10^−1^ mbar. High energetic particles may assist in breaking of silicon-oxygen bonds within the native oxide layer. Secondly, differences can be caused by the plasma chemistry. A higher degree of oxidation is expected at 1*10^−1^ mbar, since the plasma is thermalized^23^. Furthermore, the interaction between the plasma and background gas is expected to be increased at 1*10^−1^ mbar, because of the diffusion like propagation. Surprisingly, the pO_2_ at which plasma oxidation started was in the same order of magnitude for both total pressures. This observation is however in agreement with the observations made on plasmas ablated from an YBiO_3_ target^38^. In that case, oxidation of Y was observed quickly after ablation because of reaction with oxygen ablated from the target. At lower pO_2_, the pressure dependent degree of oxidation was explained by a pressure dependent oxygen nonstoichiometry of the target. If this mechanism is true, the degree of oxidation is indeed expected to be comparable in both total pressures. At low pO_2_, the interaction of the plasma with oxygen from the background is low in both total pressures. Therefore, the oxidation of the plasma is dominated by the oxygen present in the target, which depends only on the pO_2_. The plasma chemistry can however still be responsible for observed differences in growth behavior due to different arrival times. In the 2*10^−2^ mbar case, the plasma just started to oxidize when arriving at the substrate, while the amount of oxidation was higher in the 1*10^−1^ mbar case due to the increased arrival time. Therefore, the average amount of oxidized species arriving at the substrate was lower at 2*10^−2^ mbar compared to 1*10^−1^ mbar.

More work should be performed in order to elucidate the details of the influence of the plasma kinetics and chemistry on the growth process. This is important, since a proper choice of the total pressure can be used to tune the oxygen arriving from the plasma with respect to the oxygen arriving from the ambient. Furthermore, the velocity of the particles arriving at the substrate can be optimized in order to optimize the scavenging process.

## Conclusion

In this work, the growth mechanism of epitaxial YSZ on Si with native oxide by PLD was investigated. The possibility to control different sources of oxygen in the PLD process was exploited to control the chemistry during the scavenging of oxygen from the native oxide by YSZ. In conditions corresponding to optimum YSZ crystalline quality, silicide formation was prevented due to partial oxidation of the YSZ plasma and sufficient flux of oxygen from the ambient to the growing film. In this regime, significant regrowth of silicon oxide occurred, catalyzed by the deposited YSZ film. Thickness increase of the silicon oxide before growth had to be prevented by starting the depostion as soon as possible after reaching growth temperature. These findings show that the contributions of all sources of oxygen can and should be controlled in order to obtain reproducible YSZ growth.

## Methods

### Pulsed Laser Deposition

All films were grown in a TSST PLD chamber with *in situ* RHEED (STAIB). A 248 nm KrF laser (Coherent LPXpro) was used for ablation from a polycrystalline YSZ target. The base pressure of the PLD chamber was in the 10^−8^ mbar range. For low partial oxygen pressures, the oxygen flow was regulated with a needle valve, while the flow of Ar was regulated with a mass flow controller. The substrates were heated via laser heating. The deposition parameters are summarized in Table 1. Samples examined with *in situ* XPS were cooled down in vacuum, while the thicker samples examined with XRD were cooled down in 100 mbar O_2_.

**Table 1:** Parameters for YSZ deposition.

Substrate temperature (°C)	800
Heating rate (°C)	50
Cooling rate (°C)	20
Fluency (J/cm^2^)	1.9
Spot size (mm^2^)	2.4
Laser repetition rate (Hz)	Varied
Total background pressure (mbar)	0.02 or 0.1
Partial oxygen pressur (mbar)	Varied
Target-substrate distance (mm)	50

The conditions which were varied are described in the results section.

### Characterization and analysis

*In situ* XPS was performed with an Omicron XM-1000 monochromated Al-K*α* source, with the pass energy to the detector set to 20 eV. The angle of the surface normal with respect to the detector was 1°. The method to determine silicon oxide thickness was similar to the 5P* method described by Seah and Spencer^39^. An R_0_ value of 0.80 was determined by measuring silicon substrates with different thicknesses of thermally grown oxides. An attenuation length of photoelectrons in silicon dioxide of 3.448 nm was used^39^.

The chemistry of the YSZ plasma plume was assessed by self emission plasma spectroscopy. An Andor Shamrock 163 spectograph with a 300 lines/mm grating and an Andor iStar ICCD detector with 1024 × 1024 pixels were used to collect the data. This combination of spectrograph and detector resulted in a bandpass of 257 nm and a spectral resolution of 1.5 nm. The gate width was adjusted with the delay time after ablation, and typically kept below 2% of the delay time. The resulting images obtained with the CCD camera consisted of one axis representing the wavelenght, and the other axis representing the spatial component. In order to compare the measurements, the intensities were summed along the spatial axis. All spectra were normalized between 0 and 1 after subtracting the minimum intensity. The wavelength scale was calibrated using reference tables^29,30^. In order to obtain information about the individual oxides, sintered powder targets of ZrO_2_ and Y_2_O_3_ were examined as well. The time of arrival and velocity of the YSZ plasma plume at the substrate were measured by imaging the visible part of the plasma, i.e. without a spectrograph between the plasma and the camera.

XRD measurements were performed at a Panalytical X’pert Pro with a nonmonochromated Cu source, using a nickel filter to remove the K*β* emission. AFM was performed on a Bruker Dimension Icon in tapping mode.

## Electronic supplementary material

supplementary information
