# Supplementary material for: Growth mechanism of epitaxial YSZ on Si by Pulsed Laser Deposition
Source: Sci Rep. 2018 Apr 10;8:5774. doi: 10.1038/s41598-018-24025-7 (PMC5893595; doi:10.1038/s41598-018-24025-7)
Supplement: Supplementary file 1 — supplementary information [file 41598_2018_24025_MOESM1_ESM.pdf]

Supplementary information

Growth mechanism of epitaxial YSZ on Si by Pulsed Laser Deposition

David Dubbink[1], **Gertjan Koster**[1,*], **Guus Rijnders**[1]

[1]MESA+ Institute for Nanotechnology, University of Twente, Enschede, The Netherlands
*g.koster@utwente.nl

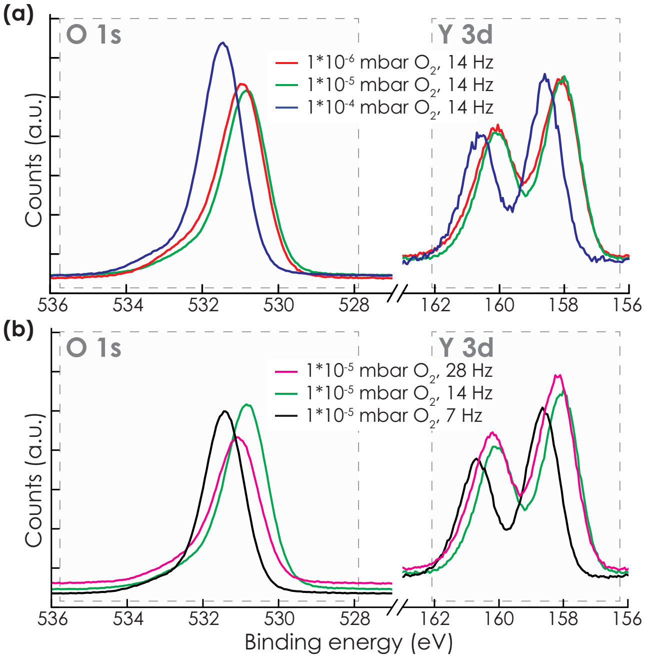

Figure S1. XPS Y3d and O1s spectra of films grown at total pressures of $2*10^{-2}$ mbar at a) different pO_2 or b) with different laser repetition rates. The spectra are from the same samples as shown in Figure 1.

In Supplementary Figure S1, the Y3d and O1s spectra corresponding to the XPS measurements of Figure 1 are shown. No clear influence of deposition conditions on the Y3d spectra was observed. Note that all peaks (Zr3d, O1s, Y3d and Si2p) of samples without silicides were shifted to higher binding energies compared to samples with silicides. The shift can be caused by the insulating character of the SiO_2 and YSZ layers[1], which are thicker in oxidizing conditions.

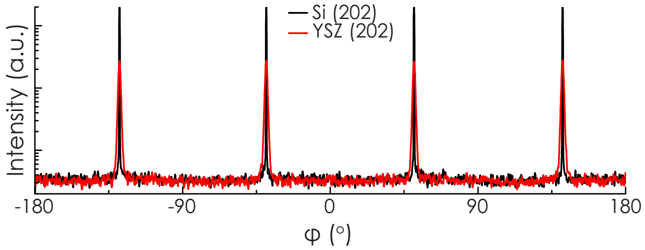

Figure S2. ϕ-scans of the Si (202) and YSZ (202) peaks of a sample grown at 14 Hz with a pO_2 of $2.5*10_3$ mbar in 0.02 mbar Ar. The [100] axes of Si and YSZ are parallel.

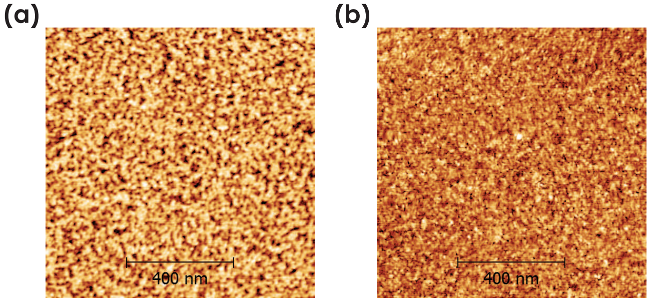

Figure S3. AFM images of films grown in pO_2 of a) $1*10^{-6}$ and b) $5*10^{-3}$ mbar, at a total pressure of $2*10^{-2}$ mbar. These images are typical for growth at pO_2 were respectively silicides are formed or formation of silicides is prevented. In conditions were silicide formation was avoided, the surface was smoother compared to conditions were silicide formation occurred (the peak-to-peak roughnesses were respectively 0.8 and 1.5 nm).

Supplementary Figure S3 shows typical AFM images of 6 nm thick films grown in conditions were silicides formed, as well as an image of a film grown in optimized conditions. The surface of the film with silicides was grainy and had an RMS of 0.39 nm (peak-to-peak roughness 1.5 nm). The film grown in optimized conditions was much smoother, and had an RMS of 0.18 nm (peak-to-peak roughness 0.8 nm).

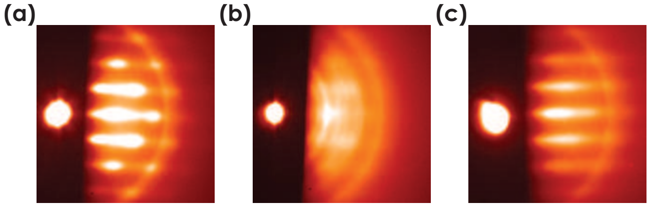

Figure S4. a) RHEED image of a film grown at a pO_2 of $1*10^{-6}$ mbar in a total pressure of $2*10^{-2}$ mbar Ar, where silicides were observed with XPS. b) RHEED image grown in the same conditions, but after cooling down in O_2. c) RHEED image of a film grown at $5*10^{-3}$ mbar O_2 in a total pressure of $2*10^{-2}$ mbar Ar, showing the streaks consistent with a flat surface. No changes were observed in the pattern when more O_2 was added to the system.

A similar observation was made by RHEED (see Supplementary Fig. S4). The film grown in optimized conditions had a streaky pattern, indicating a flat film, while the film grown in oxygen deficient conditions had a spotty pattern, indicating island formation. Although both patterns indicate epitaxial grown films, a big difference was observed when O_2 was added directly after growth. Patterns of films grown in optimized conditions remained streaky. However, rings indicating polycrystallinity appeared in the film grown in the more reducing conditions. In the XPS measurements performed on this film, no features indicating silicides were visible anymore, while the ratio of SiO_2 with respect to silicates/SiO_x increased (see Supplementary Fig. S5).

The crystalline quality of the YSZ was low in conditions where silicide formation was observed. Silicides form at the YSZ-Si interface[2], and are not necessarely good templates for YSZ crystallization. The roughness observed with AFM could therefore be caused by the formation of dewetted YSZ alternated with regions where zirconium silicide phases are exposed.

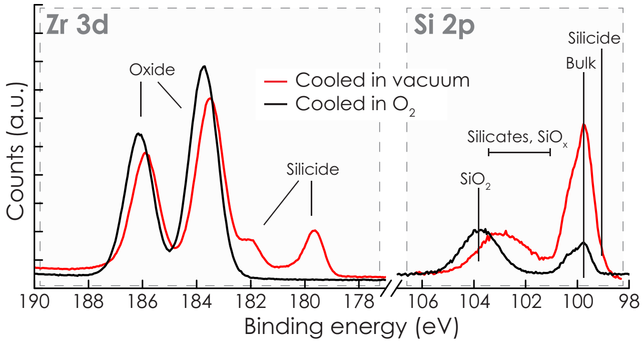

Figure S5. XPS Zr3d and Si2p spectra of two films grown at $1*10^{-6}$ mbar O_2 in a total pressure of $2*10^{-2}$ mbar Ar. The features indicating silicide formation were not visible in the sample which was cooled down in O_2.

Existance of exposed silicide phases is in agreement with the observations made when those films were exposed to oxygen. XPS showed that silicides were not stable in oxygen environment, and tranformed to SiO_2 and YSZ. Formation of polycrystalline phases was observed with RHEED. Probably, crystallization of YSZ from the silicide phase occured simultaneously with the formation of amorphous SiO_2, in regions visible with RHEED. The presence of exposed polycrystalline YSZ and amorphous SiO_2 results in a bad template for further YSZ growth. This process can still occur in partial oxygen pressures above which silicide formation was detected with XPS. Small amounts of silicides can be transformed to YSZ and SiO_2 during cool down before being measured with XPS, and have a similar effect on the quality of the template. This explains, for example, why an optimum quality was not reached at a pO_2 of $1*10^{-4}$ in $2*10^{-2}$ mbar Ar yet, while no silicides were measured anymore.

References

1. Abe, Y. *et al.* Effect of Oxide Charge Trapping on X-ray Photoelectron Spectroscopy of $HfO_2/SiO_2/Si$ Structures. *Jpn. J. Appl. Phys.* **48**, 041201 (2009). URL http://stacks.iop.org/1347-4065/48/041201. DOI 10.1143/JJAP.48.041201.

2. de Coux, P. *et al.* Mechanisms of epitaxy and defects at the interface in ultrathin YSZ films on Si(001). *CrystEngComm* **14**, 7851 (2012). URL http://xlink.rsc.org/?DOI=c2ce26155c. DOI 10.1039/c2ce26155c.
